# Genetic Variants Associated with Fluoropyrimidine-Induced Toxicity in Real-World Patients After Pre-Emptive *DPYD* Pharmacogenetic Testing

**DOI:** 10.3390/ph19030460

**Published:** 2026-03-11

**Authors:** María Rodríguez-García, Sara Salvador-Martín, Mariam Stephanie Rojas Piedra, Adrián Bravo, María Vidal, Carolina Figueras Gutierrez, Joan Maurel, Luis A. López-Fernández, Mercè Brunet

**Affiliations:** 1 Biochemistry and Molecular Genetics Department, Biomedical Diagnostic Center (CDB), Hospital Clínic of Barcelona, 08036 Barcelona, Spain; mrodriguezg@clinic.cat; 2 Institut d’Investigacions Biomèdiques August Pi i Sunyer IDIBAPS, 08036 Barcelona, Spain; 3 Faculty of Medicine and Health Science, University of Barcelona, 08036 Barcelona, Spain; 4 Pharmacy Department, Hospital General Universitario Gregorio Marañón, Instituto de Investigación Sanitaria Gregorio Marañón, 28007 Madrid, Spain; 5 Medical Oncology Department, Hospital Clínic of Barcelona, 08036 Barcelona, Spain; 6 Biochemical Department, Hospital General Universitario Gregorio Marañón, 28007 Madrid, Spain; 7 Translational Genomics and Targeted Therapies in Solid Tumors, Agustí Pi i Sunyer Biomedical Research Institute (IDIBAPS), 08036 Barcelona, Spain; 8 CIBEREHD, Network Centre for Hepatic and Digestive Diseases, National Spanish Health Institute Carlos III (ISCIII), 28029 Madrid, Spain; 9 Pharmacology and Toxicology, Biochemistry and Molecular Genetics, Biomedical Diagnostic Center (CDB), Hospital Clinic of Barcelona, University of Barcelona, 08036 Barcelona, Spain

**Keywords:** *DPYD* pre-emptive pharmacogenetics, *DPYD* new variants, fluoropyrimidine toxicity

## Abstract

**Background/Objectives**: As a treatment, fluoropyrimidines are often associated with early moderate-to-severe toxicity. Although pre-emptive *DPYD* genotyping enables genotype-guided dosing, significant adverse events still occur in patients classified as *DPYD* wild-type (WT). The aim of this study was to identify *DPYD* variants and evaluate the contributions of *TYMS*, *ENOSF1*, *CDH4,* and *CDA* variants to fluoropyrimidine toxicity. **Methods**: A total of 256 European ancestry patients (aged ≥ 18 years, had completed ≥6 cycles of chemotherapy, and were WT for the *DPYD* variants routinely tested for) underwent genotyping for *TYMS*, *ENOSF1*, *CDH4,* and *CDA* variants, and full *DPYD* exon sequencing was performed in 56 of these patients. Toxicity was defined as fluoropyrimidine-related adverse events requiring a dose reduction. Multivariable models were adjusted for sex, fluoropyrimidine type, and the Charlson Comorbidity Index, and time-to-event was assessed using the Kaplan–Meier/Cox proportional hazards models. **Results**: A subgroup of 117 patients experienced toxicity requiring a dose reduction. The most frequent events were asthenia, gastrointestinal toxicity, hand–foot syndrome, and haematological toxicity. The *ENOSF1* rs2612091 C allele was associated with fluoropyrimidine withdrawal and a shorter time to dose reduction. In the patients treated with 5-fluorouracil, *TYMS* rs11280056 was associated with toxicity. *DPYD* exon sequencing identified thirteen variants, nine of which were more prevalent in the toxicity group. These included a canonical splice site (c.150+1G>A) and a stop-gained (c.1863G>A) variant, which is predicted to result in loss of function. **Conclusions**: In real-world practice, despite undergoing standard *DPYD* genotyping, *DPYD* WT patients receiving a full dose of fluoropyrimidines develop clinically relevant toxicity. The presence of rare *DPYD* variants and associated genes (*ENOSF1* and *TYMS*) suggests that broader, prospectively validated pharmacogenetic strategies may improve toxicity prevention.

## 1. Introduction

Fluoropyrimidines, including 5-fluorouracil (5-FU) and its oral prodrug capecitabine, are widely used in the treatment of various solid tumours, such as colorectal, anal canal, oesophageal, gastric, breast, and head and neck cancers. Up to approximately 30% of patients experience moderate-to-severe toxicity during the early stages of treatment with fluoropyrimidines [1], often due to reduced activity of the key metabolic enzyme dihydropyrimidine dehydrogenase (DPD), which is encoded by the *DPYD* gene and plays a central role in the metabolism of fluoropyrimidines into inactive metabolites. *DPYD* gene variants are associated with reduced DPD activity, which can lead to severe adverse reactions to fluoropyrimidines. In routine clinical practice, stratifying patients based on their *DPYD* genotype may help prevent such toxicities [2,3,4,5,6].

The *DPYD* variants associated with decreased DPD function include the loss-of-function alleles rs3918290 (DPYD*2A; c.1905+1G>A; IVS14+1G>A) and *DPYD* rs55886062 (DPYD*13, c.1679T>G; p.I560S), and the reduced-activity variants *DPYD* rs67376798 (c.2846A>T; p.D949V), rs75017182 (*DPYD* c.1129-5923C>G) included in haplotype B3 with rs56038477 (c.1236G>A; p.E412E). These variants partially account for the interindividual differences in fluoropyrimidine toxicity. The European Medicines Agency (EMA) recommends testing for DPD deficiency prior to initiating treatment with fluoropyrimidines [7]; in addition, the Clinical Pharmacogenetics Implementation Consortium (CPIC) and the Dutch Pharmacogenetics Working Group (DPWG) have published dosing guidelines for 5-FU and capecitabine based on *DPYD* genotype. Patients with a gene activity score of 0–0.5 are advised to avoid fluoropyrimidines, while those with a score of 1–1.5 are recommended to initiate treatment at 50% of the standard dose of 5-FU or capecitabine [8,9,10,11,12]. The current CPIC guideline also notes that patients with the c.2846A>T/c.2846A>T genotype (activity score = 1) may require a >50% reduction in the starting dose [13].

Pre-emptive *DPYD* genotyping has been incorporated into the Spanish National Health System portfolio of genomic services since 2023. However, despite genotype-guided dosing, adverse events still occur. Approximately 25% of patients with a wild-type (WT) *DPYD* genotype (where WT refers to the absence of decreased-function *DPYD* variants included in the most commonly used guideline-based panels) receiving standard initial doses of 5-FU or capecitabine experience fluoropyrimidine-related toxicity [8,14]. This is because the most commonly used guideline-based panels may not capture rare, novel, or not yet functionally characterised variations that could still influence toxicity risk.

Beyond *DPYD*, several additional genes contribute to the risk of fluoropyrimidine-induced toxicity and may improve risk prediction. Although DPD deficiency is the main determinant of impaired metabolism and severe adverse reactions (ClinGen’s Pharmacogenomics curation rates the *DPYD*–fluorouracil/capecitabine association at the highest level of evidence), a substantial proportion of toxicity remains unexplained by *DPYD* variants alone [15].

*TYMS*, which encodes the direct target of fluoropyrimidines, has been associated with toxicity through polymorphisms in its 3′-UTR and 5′-VNTR regions, particularly for hand–foot syndrome [16]. However, growing evidence suggests that these associations may be mediated by regulatory effects of the adjacent *ENOSF1* gene, with intronic *ENOSF1* variants (e.g., rs2612091) showing strong independent links to toxicity [15].

*CDA* variants have been associated with increased risk of capecitabine-related toxicities such as diarrhoea, independently of *DPYD* genotype [17]. Finally, *CDH4* has emerged from recent pharmacogenomic burden analyses as a candidate gene, with rare functional variants potentially refining toxicity risk prediction when combined with established pharmacogenetic markers [18].

The aim of this study was to identify new genetic *DPYD* variants as well as the potential role of *TYMS*, *ENOSF1*, *CDA*, and *CDH4* genetic variants associated with fluoropyrimidine toxicity in patients who have a WT *DPYD* genotype who are receiving the full starting dose.

## 2. Results

### 2.1. Patients

**Figure 1:**
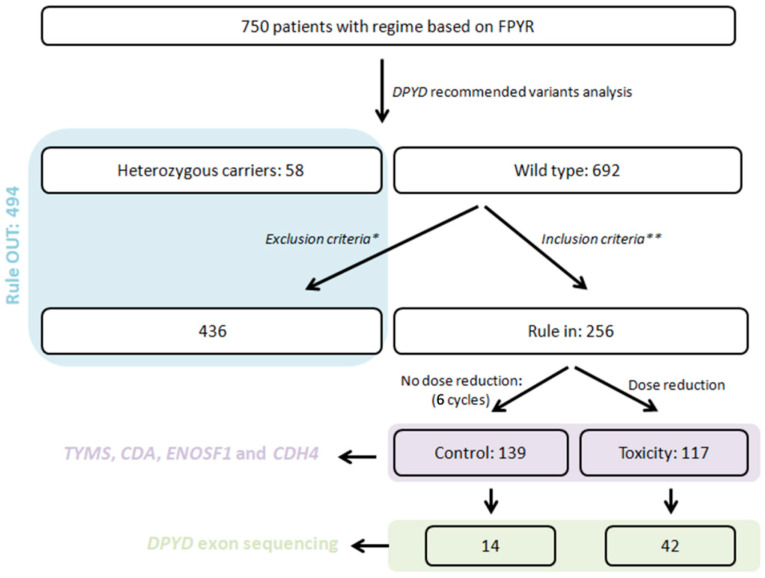
Study Flow Diagram: Patient Selection and Group Assignment. A total of 750 patients who had been treated with a fluoropyrimidine-based regimen were screened. Pre-emptive *DPYD* polymorphism analysis for the recommended variants was performed. Of the eligible patients, 256 were included and classified according to whether they required a dose reduction during the first six cycles of chemotherapy: a control group (no dose reduction; n = 139) and a toxicity group (dose reduction; n = 117). In the included cohort, *TYMS*, *CDA*, *ENOSF1* and *CDH4* variants were assessed, and *DPYD* exon sequencing was performed in a subset of patients (control group: n = 14; toxicity group: n = 42). * Patients were excluded if they discontinued treatment, continued therapy at another institution, or died during the follow-up period. ** The study included patients over 18 years of age who had completed at least six cycles of therapy and were wild-type for the analysed variants.

We summarise the characteristics of the included cohort and compare the clinical features of the toxicity and control groups (Figure 1). Here, we also describe the main fluoropyrimidine-related adverse events observed overall and by cancer type, and report the frequency of routinely tested *DPYD* variants in the study population.

Of 750 patients, a total of 256 patients, 124 (48.4%) men and 132 (51.6%) women, aged 18 to 88 years, were included after applying the inclusion and exclusion criteria (Table 1).

The most common cancer type was colorectal cancer (51.6%), followed by breast cancer (15.2%). The toxicity group contained 117 patients, while the control group comprised 139 patients. No major differences were observed in either group in terms of sex, age, type of fluoropyrimidine, regimen, type of cancer, Charlson Comorbidity Index, or line of treatment (Table 1).

All 117 patients in the toxicity group initially presented with moderate toxicity, which required dose reduction. The most frequently reported adverse events in the overall toxicity group were asthenia, gastrointestinal toxicity, hand–foot syndrome, and haematological toxicity. After stratifying by cancer type, in those with colorectal cancer (n = 66), the most common events observed were asthenia and gastrointestinal toxicity; meanwhile, in those with breast cancer, hand–foot syndrome predominated, with lower rates of asthenia (Table S2).

In relation to the genetic variants currently analysed in daily clinical practice of 750 patients, 22 patients (2.9%) were heterozygous carriers of the rs75017182 (*DPYD* c.1129-5923C>G) included in haplotype B3 with rs56038477 (c.1236G>A; p.E412E) variant, 13 patients (1.73%) were heterozygous carriers of the rs3918290 DPYD*2A (c.1905+1G>A; IVS14+1G>A) and 8 (1.06%) patients were carriers of the *DPYD* rs67376798 (c.2846A>T; p.D949V)). No homozygous carriers of any of these variants were identified. All patients were WT for the *DPYD* rs55886062 (DPYD*13, c.1679T>G; p.I560S).

### 2.2. Association of TYMS, ENOSF1, CDH4, and CDA with Toxicity and Dose Reduction

In this sub-analysis, we evaluated the relationship between selected genetic variants in *TYMS*, *ENOSF1*, *CDH4*, and *CDA* and the occurrence of fluoropyrimidine-related toxicity or dose modification events. The frequency of variants in *TYMS*, *ENOSF1*, *CDH4*, and *CDA* in toxicity or dose reduction groups and controls was analysed.

The association of the selected genetic variants was analysed with additive, dominant, and recessive models (Table S3); univariate and multivariate analyses were also performed.

The *ENOSF1* rs2612091 C variant was associated with toxicity in an additive univariate analysis (*p* value 0.041) as well as in a multivariate analysis considering sex, type of fluoropyrimidine, Charlson Comorbidity Index, regimen and indication: TT (ref) vs. CC (OR 2.063; 95% IC 1.000–4.257, *p* value 0.049) (Table 2).

When analysing the cohort of patients disaggregated by type 5-FU that carried (Tables S4 and S5), an association was observed for the patients treated with 5-FU that carried the *TYMS* rs11280056 variant in a dominant model in both univariate and multivariate analyses (*p* value 0.042 and *p* value 0.042, respectively) (Table 2).

**Figure 2:**
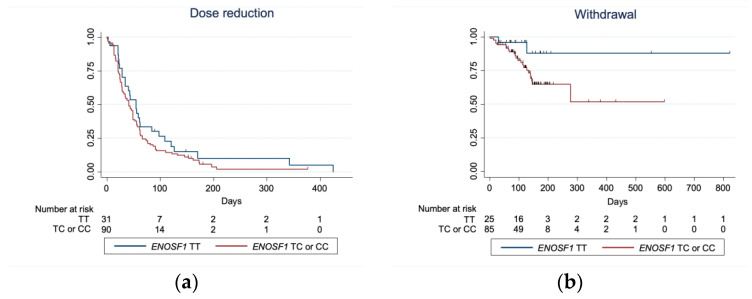
Kaplan–Meier analyses dose reduction and treatment withdrawal by *ENOSF1* rs2612091 genotype. (**a**) Kaplan–Meier curve illustrating the association between the *ENOSF1* rs2612091 variant and the time to onset of toxicity or fluoropyrimidine dose reduction. (**b**) Kaplan–Meier curve showing the relationship between the same variant and time to treatment withdrawal.

Because toxicity, treatment delays, and withdrawals often affect the cumulative dose, we performed a Kaplan–Meier analysis to assess whether the significant associations identified were linked to the time to onset of toxicity or fluoropyrimidine dose reduction (Figure 2a). The rs2612091-C allele in the *ENOSF1* gene was associated with a shorter time from treatment initiation to the occurrence of an adverse event or dose reduction (*p* value = 0.047, OR 1.551, 95% IC (1.005–2.393) with sex, fluoropyrimidine type and Charlson Comorbidity Index as covariates.

### 2.3. Association of TYMS, ENOSF1, CDH4, and CDA with Drug Withdrawal

We examined whether the selected genetic variants were associated with fluoropyrimidine treatment withdrawal as a specific clinical end-point. The same sequence of analyses was performed, taking withdrawal of fluoropyrimidine treatment as the end-point (Table S6). The *ENOSF1* rs2612091 C variant was associated with withdrawal in an additive univariate analysis (*p* value 0.008) as well as in a multivariate analysis considering sex, type of fluoropyrimidine, Charlson Comorbidity Index, regimen and indication: TT (ref) vs. TC (OR 4.799; 95% IC 1.055–21.825, *p* value 0.042) and TT (ref) vs. CC (OR 7.040, 95% IC 1.434–34.564, *p* value 0.016) (Table 3). This association was maintained in a dominant model (*p* value 0.011) even after multiple analyses: TT (ref) vs. TC/CC (OR 5.441, 95% IC 1.239–23.885, *p* value 0.025). When analysing the cohort of patients disaggregated by type of fluoropyrimidine (Tables S7 and S8), an association was observed for the patients treated with 5-FU in both additive and dominant models (*p* value 0.013 and 0.010, respectively) (Table 3).

A Kaplan–Meier curve was created to assess the association between the rs2612091 variant in the *ENOSF1* gene and the time to treatment withdrawal. The curve illustrates an increased risk of withdrawal over time in patients carrying the C allele (*p* = 0.048; OR 4.350 (1.014–18.663)) with sex, fluoropyrimidine type, Charlson Comorbidity Index, regimen and indication as covariates (Figure 2b).

### 2.4. Analysis of DPYD Exon Sequences

We investigated *DPYD* exonic variation by performing full sequencing of all 23 coding regions to explore potential associations between rare variants and fluoropyrimidine-related toxicity. The whole exonic regions of the *DPYD* gene were fully sequenced in 42 patients from the toxicity group and 14 from the control group. A total of 13 genetic variants were identified among the sequenced patients (Table 4), 9 of which occurred more frequently in the toxicity group than in the control group (c.85C>T, c.150+1G>A, c.218T>G, c.515G>A, c.775A>G, c.1218G>A, c.1627A>G, c.1863G>A, and c.2194G>A). Sub-analysis for exon variants was underpowered, and statistical analysis was not undertaken.

Most of the variants detected were only seen in one patient. For the more common variants, the minor allele frequency (MAF) was calculated for the control and toxicity groups. The SNV c.2194G>A (DPYD*6) was absent in the control group, while its frequency in the toxicity group was 9%.

Two of the variants detected have the potential to generate non-functional DPD proteins. Specifically, the c.150+1G>C variant affects a canonical splice site, which is essential for the proper splicing of pre-mRNA and the generation of functional mRNA and protein. The genomic variant c.1863G>A p.Trp621Ter rs1057516388 on the *DPYD* gene is a nonsense mutation that results in the premature termination of the protein, leading to a truncated and likely non-functional enzyme.

**Figure 3:**
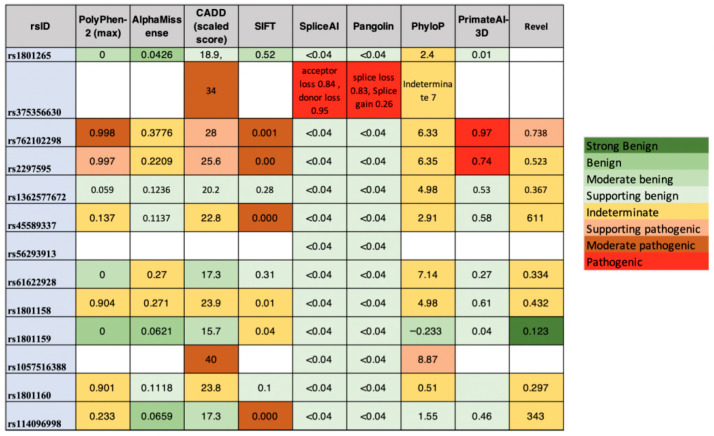
In silico prediction of functional impact and splice alterations identified variants. Summary of computational predictions generated for all variants detected in the study. SpliceAI scores for donor and acceptor gain/loss events are shown, together with additional pathogenicity and conservation metrics including PolyPhen-2 (max), AlphaMissense, CADD (scaled score), SIFT, PhyloP, PrimateAI-3D, REVEL, and Pangolin. Variants are classified according to their predicted impact severity, ranging from benign to pathogenic, based on integrated outputs from these algorithms.

SpliceAI was used to predict the splicing alterations caused by the variants found (Figure 3). This tool also provides information from other predictive models such as PolyPhen (max), AlphaMissense, CADD, SIFT, PhyloP, PrimateAI-3, REVEL, and Pangolin.

### 2.5. DPD Tertiary Structure Changes Caused by Variants c.150+1G>A and c.1863G>A

We examined the functional consequences of rare *DPYD* variants by characterising their predicted impact on mRNA splicing, protein truncation, and DPD structural integrity.

The *DPYD* gene variant c.150+1G>A variant in the *DPYD* gene is a splicing mutation. This specific nucleotide substitution, located at the canonical splice donor site of an intron, disrupts normal pre-mRNA splicing, probably leading to the complete skipping of exon 2 from the mature *DPYD* messenger RNA.

The c.1863G>A variant changes a tryptophan (Trp) codon at amino acid position 621 to a premature stop codon (p.Trp621Ter). Consequently, this results in a truncated DPD protein that lacks all amino acids from position 621 onwards, which includes part of domain 3 (four [4Fe-4S] iron–sulphur cluster), and fully domains 4 (substrate-binding) and 5 (dimerisation). This significant amount of sequence that is lost very likely includes essential functional domains critical for the protein’s proper activity, leading to a severely impaired or non-functional DPD enzyme.

**Figure 4:**
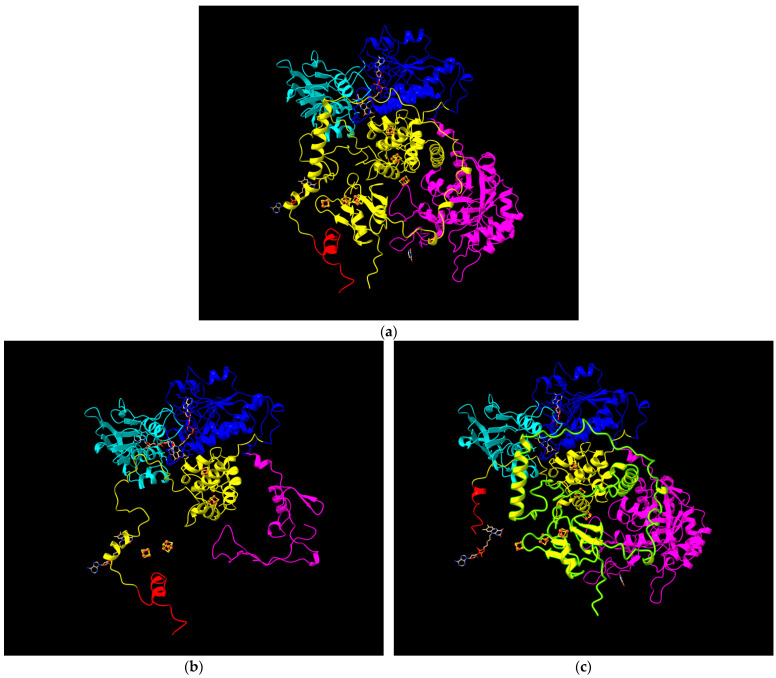
Structural impact of *DPYD* pathogenic variants affecting splicing or introducing a premature stop codon. (**a**) Consensus DPD structure, showing intact domain organization; (**b**) splice-altering c.150+1G>A variant, predicted to cause exon 2 skipping and resulting in an N-terminally altered DPD protein lacking correct folding and early structural elements required for proper domain assembly; (**c**) premature stop variant c.1863G>A (p.Trp621Ter), generating a truncated protein that lacks part of the [4Fe-4S]–containing domain and completely loses the substrate-binding and dimerization domains, severely compromising DPD structural integrity. DPD domains shown: Domain V (red), iron–sulphur cluster (FeSFeS, yellow), NADPH DII (blue), NADPH DIII (light blue), and FMN/5-FU (magenta).

Structural simulations were performed to visualise the tertiary structure of DPD in the presence of these mutations. Specifically, using RaptorX for protein structure prediction and Chimera for visualisation, we have generated models illustrating the predicted impact of the c.150+1G>A and c.1863G>A variants on the DPD protein (Figure 4).

## 3. Discussion

Using data from real-world clinical practice, this study showed that, despite pre-emptive *DPYD* genotyping to guide the initial fluoropyrimidine dose selection, 38% of patients with *DPYD* wild-type status experienced adverse events, leading to dose reduction or treatment discontinuation. Our exploratory results suggest that multiple genetic approaches, including *DPYD* sequencing and genotype of *TYMS* and *ENOSF1* variants, may help identify a subset of patients who do not carry any of the currently recommended *DPYD* variants who may be at risk of fluoropyrimidine-related toxicity. Nine *DPYD* variants were identified with a higher frequency in the toxicity group (c.85C>T, c.150+1G>A, c.218T>G, c.515G>A, c.775A>G, c.1218G>A, c.1627A>G, c.1863G>A, and c.2194G>A) than in the control group. Although statistical analyses were not conducted due to limited sample size, one of them, c.1863G>A is identified for the first time in a patient with toxicity/dose reduction and withdrawal from fluoropyrimidines and 5-FU.

Two genetic variants in *DPYD* could explain the observed toxicity in carrier patients: c.151+1G>T and c.1863G>A. Alterations in splice sites can lead to aberrant splicing, potentially resulting in the inclusion or exclusion of exons, the retention of introns, or the use of cryptic splice sites, all of which can disrupt the normal function of the protein. Exon 2 encodes part of the FAD-binding domain, which is essential for electron transfer from NADPH to the pyrimidine substrate. Loss of this region disrupts proper folding and cofactor binding, leading to complete loss of DPD enzymatic activity [19]. Computational evidence derived from CADD, SpliceAI and Pangolin supports the predicted effect of the variant. According to American College of Medical Genetics and Genomics (ACMG) and Association for Molecular Pathology (AMP) criteria, this variant has been classified as likely pathogenic for one MedGen condition and of uncertain significance for three MedGen conditions; among these, the most severe classification is likely pathogenic for DPD deficiency, for which the variant met the following ACMG criteria: PVS1 (null variant in a gene where LOF is a known mechanism of disease), PM2 (absent of controls in population databases) (downgraded to evidence supporting) [20]. Functional studies and case reports, some of which were authored by our group, have consistently demonstrated that splice-site mutations at positions such as c.151+1G>T are pathogenic and should be considered high-risk alleles for DPD deficiency. These include the variants c.1905+1G>A [21], c.321+1G>A [22], c.2242+1G>T [23], c.1740+1G>T, IVS11+1G>T [24] and c.763-2A>G [25]. Structural exon deletions—including exons 4 [26], 6 [27], 9–10 [28], 12 [29], 11 [30] and 14–16 [29] are also linked to DPD deficiency and fluoropyrimidine toxicity. These data strongly support c.150+1G>C as a loss-of-function variant. However, this is the first association of c-151+1G>T with fluoropyrimidine-based toxicity in a real-life patient.

The genomic variant c.1863G>A p.Trp621Ter rs1057516388 in the *DPYD* gene is a nonsense mutation that results in the premature protein termination of the protein, leading to a truncated and likely non-functional enzyme. A premature stop codon at amino acid 621 in the DPD protein results in truncation of the C-terminal region, disrupting structural domains essential for enzymatic function. Specifically, translation halts within domain 3 (residues ~523–738), which mediates FAD binding and catalytic activity, and fully eliminates domains 4 and 5, which coordinate [4Fe–4S] clusters and FMN/FAD cofactors required for electron transfer [31,32]. This loss abolishes the activity of DPD, impairing the initiation of pyrimidine catabolism. As a result, 5-fluorouracil (5-FU) cannot be degraded efficiently, leading to drug accumulation and a significantly increased risk of severe, potentially life-threatening toxicity during chemotherapy. No direct functional studies exist for p.Trp621Ter, but premature stop variants like p.P633Qfs (DPYD*3) show complete loss of activity (CPIC level 1A) [33]. Therefore, p.Trp621Ter is also highly likely to abolish DPD function. This variant has been identified in a patient with a psychiatric disorder, suggesting a potential role in neuropsychiatric conditions [8]. Loss of DPD activity produces two clinically relevant and interconnected phenomena. On one hand, endogenous toxicity from elevated levels of uracil/thymine leads to neurological/neuropsychiatric disorders; on the other hand, exogenous toxicity from the inability to eliminate fluoropyrimidine drugs causes life-threatening chemotoxicity. However, the exact clinical significance of this variant in the context of psychiatric disorders remains to be fully elucidated. The variant c.1863G>A p.Trp621Ter rs1057516388 on the *DPYD* gene is a stop-gained mutation. It is present in the general population with a frequency of 0.00043% according to gnomAD, and there are no reported homozygous individuals for this variant in the database. This is the first description of a real patient carrying this mutation who exhibited fluoropyrimidine-based toxicity. Computational evidence provided by CADD and PhyloP analyses supports the predicted effect of the variant. This variant has been classified, according to ACMG/AMP criteria, as pathogenic for one MedGen condition and of uncertain significance for three MedGen conditions; the most severe classification, pathogenic, has been assigned for DPD deficiency, for which the variant met the following ACMG criteria: PVS1, PS1 (same amino acid change as a previously established pathogenic variant regardless of nucleotide change), PM2 (downgraded to evidence supporting) [20].

Other *DPYD* variants were identified exclusively in the toxicity group or with a higher frequency in the toxicity group compared to the control group. The most promising biomarker for minimising fluoropyrimidine-induced toxicity is c.2194G>A (p.Val732Ile, DPYD*6). Although functional studies have shown that this variant does not affect DPD in vitro activity [34], a recent meta-analysis confirmed that the pooled OR for overall toxicity in patients with the A allele was elevated by 1.73 times compared to those with the GG genotype (95% CI 1.44–2.07) [35]. Multiple studies have shown that DPYD*6 carriers exhibit a higher incidence of grade ≥ 3 adverse drug reactions (ADRs), particularly gastrointestinal and haematological toxicities, compared to wild-type individuals, but the risk increase is less pronounced than with the classic high-risk alleles [36,37,38]. This toxicity appears to be more common in women than in men, in individuals carrying the DPYD*6 allele [39]. Recently, a lower dihydrouracil-to-uracil ratio (UH2/U) has been observed in DPYD*6 carriers [39]. Recognising the clinical significance of this polymorphism, the Italian Society of Oncology guidelines include the prior determination of DPYD*6 in their recommended *DPYD* testing panels. For patients heterozygous for the variant, a 15% reduction in fluoropyrimidine dosage is recommended, while for those who are homozygous for the variant, a 30% dose reduction is advised [40]. In parallel, the contradictory findings for the c.775A>G variant—found more frequently in the toxicity group despite being commonly classified as having normal activity—suggest that it should instead be considered a variant of uncertain function. In silico modelling predicts that the c.775A>G (p.K259E) variant may impair protein function by altering the local electrostatic environment and disrupting interactions with critical cofactors [41]. Furthermore, in a retrospective cohort of patients treated with fluoropyrimidines, the c.775A>G variant was observed in a heterozygous state exclusively among individuals classified as *DPYD* poor metabolizers [42].

Regarding the frequency of rare variants, a previous study reported a higher proportion of *DPYD* rare variants in patients who developed grade ≥ 3 toxicity under fluoropyrimidine-based treatment compared to control patients [43]. In line with this observation, in our study, seven of the identified *DPYD* variants were rare (MAF < 0.1), and six of them were found in patients in the toxicity group. This reinforces the notion that rare *DPYD* variants are risk factors for fluoropyrimidine-induced toxicity.

Our results also showed that the *ENOSF1* rs2612091 C variant was associated with global toxicity, and a trend was observed with time to withdrawal of fluoropyrimidine-based treatment. This intronic variant has been associated with an increased risk of severe fluoropyrimidine toxicity, and most notably severe hand–foot syndrome (HFS), independent of *DPYD* status. The rs2612091 variant confers an approximately 1.6-fold increased risk per allele for severe HFS in patients treated with capecitabine or 5-fluorouracil, as demonstrated in large candidate gene studies and meta-analyses. This association is independent of the well-characterised *DPYD* variants and persists even in patients who have undergone *DPYD* genotyping and to whom genotype-guided dosing has been administered [17,44]. However, the clinical utility of *ENOSF1* genotyping remains limited. The effect size, while statistically significant, is modest compared to that for *DPYD* variants, and current pharmacogenetic guidelines do not recommend routine *ENOSF1* testing for dose adjustment or toxicity prevention. The predictive value of *ENOSF1* variants is primarily associated with HFS rather than global severe toxicity, and no dosing recommendations are established based on *ENOSF1* genotype [44].

*TYMS* gene variants, particularly the 5′-untranslated region (5′-UTR) variable number tandem repeat (VNTR) polymorphism (2R/2R genotype) and the 3′-UTR 6-bp insertion/deletion, are associated with an increased risk of severe toxicity from fluoropyrimidine chemotherapy. Patients with the 2R/2R genotype or the 2R/3R genotype have a higher incidence of grade 3–4 adverse events compared to those with the 3R/3R genotype, with reported rates of severe toxicity up to 43% for 2R/2R, 18% for 2R/3R, and 3% for 3R/3R in colorectal cancer patients receiving 5-FU-based chemotherapy. The risk is particularly notable for gastrointestinal toxicity, including diarrhoea, and for hand–foot syndrome (HFS) [45].

However, recent large-scale and meta-analytic studies have indicated that the association between *TYMS* variants and global fluoropyrimidine toxicity is largely explained by linkage disequilibrium with *ENOSF1* variants, especially rs2612091. *ENOSF1* and *TYMS* variants independently and additively increase the risk of severe HFS, with each risk allele conferring a 1.3–1.6-fold increased risk; patients homozygous for both risk alleles have up to a 3-fold higher risk of severe HFS [45]. In multivariate analyses, the effect of *TYMS* variants on toxicity is attenuated when *ENOSF1* status is included, suggesting that *ENOSF1* may be the primary driver of this association [15].

In patients who have already undergone *DPYD* genotyping, *TYMS* and *ENOSF1* variants remain independent risk factors for severe hand-foot syndrome and, to a lesser extent, global toxicity. However, current clinical guidelines do not recommend routine *TYMS* or *ENOSF1* genotyping for dose adjustment, as its predictive value is lower than for *DPYD* variants, and no consensus on dose modification exists [44]. These findings support that further studies with larger cohorts and treatment guidance based on these variants are necessary to better evaluate their impact in determining toxicity factors.

We wish to emphasise that our results were obtained and evaluated in patients treated in a real-world clinical setting, rather than in highly selected populations typically used in clinical trials.

In our cohort, genetic variants that may influence the observed toxicity profile. Other potential confounders—including sex, type of fluoropyrimidine (capecitabine or fluorouracil), comorbidities assessed with the Charlson score, line of treatment, and regimen—were evaluated and showed no significant association with toxicity. Importantly, even though initial dose adjustments were made according to the CARG assessment, these genetic associations with toxicity remained evident.

This study has several limitations. Firstly, the cohort was derived from routine care in a specific clinical setting and was limited to a European ancestry population. This may restrict the applicability of these findings to populations with different genetic architectures and allele frequencies. Secondly, although toxicity was assessed using standard clinical criteria, concomitant non-antineoplastic medications that could reasonably contribute to, exacerbate, or mimic fluoropyrimidine-related adverse events were not systematically accounted for. In contrast, concomitant antineoplastic therapies were captured as part of the treatment regimen. Therefore, residual confounding in toxicity attribution cannot be excluded.

## 4. Materials and Methods

### 4.1. Design

This was an observational, longitudinal, and retrospective study conducted in two centres in Spain.

### 4.2. Patients and Ethics

A total of 750 whole blood samples from 750 patients (one sample per patient) treated at the Oncology Department of the Hospital Clínic of Barcelona, who were referred for *DPYD* gene polymorphism analysis, were received at the Pharmacology Laboratory of Hospital Clínic of Barcelona between March 2020 and December 2022. All patients had a diagnosis of solid tumour and were treated with a fluoropyrimidine-based regimen. Pre-emptive *DPYD* polymorphism analysis for the recommended variants was performed according to CPIC [12] and SEFF [9] consensus guidelines. The study included patients over 18 years of age who had completed at least six cycles of therapy and who were wild-type for the routinely analysed variants DPYD*2A (rs3918290, c.1905 + 1G>A, IVS14 + 1G>A), DPYD*13 (rs55886062, c.1679T>G, I560S), *DPYD* c.2846A>T (rs67376798, D949V), and *DPYD* c.1129-5923C>G (rs75017182; haplotype B3) [12]. Patients carrying any of these variants were excluded from the study. Patients were also excluded if they discontinued treatment, continued therapy at another institution, or died during the follow-up period Figure 1.

This study was approved by the Ethics Committee of Hospital Clínic (HCB/2019/0255) and conducted in accordance with the World Medical Association Declaration of Helsinki and Spanish legislation. Written informed consent for the pharmacogenetic study was obtained from all patients.

The following variables were collected from electronic clinical records: sex, age, cancer type, type of fluoropyrimidine administered, treatment regimen, line of chemotherapy, comorbidities (assessed using the Charlson Comorbidity Index), concomitant antineoplastic therapies (included within the treatment regimen), adverse events related to fluoropyrimidine treatment, and the corresponding toxicity severity grade of toxicity (according to the National Cancer Institute Common Terminology Criteria for Adverse Events, version 5.0 grade ≥ 2 or ≥3). Grade 1 asthenia was only recorded as a toxicity event when it occurred with other adverse effects. Therapeutic regimens included monotherapy (fluoropyrimidine alone, e.g., capecitabine), CAPOX (capecitabine and oxaliplatin), FOLFOX (5-fluorouracil, leucovorin, and oxaliplatin), FOLFIRI (5-fluorouracil, leucovorin, and irinotecan), FOLFIRINOX (5-fluorouracil, leucovorin, oxaliplatin, and irinotecan), cisplatin plus fluorouracil, and FLOT (5-fluorouracil, leucovorin, oxaliplatin, and docetaxel). In addition, comorbidities in the study population were assessed using the Charlson Comorbidity Index (CCI). For simplicity and relevance, the abbreviated version of the CCI was applied. Each comorbidity was assigned a weighted score according to its severity and impact on mortality, and a total score was calculated for each patient (Table S1).

Before therapy, all patients ≥ 70 years old were evaluated by a specialised nurse to assess basal comorbidities and adjust therapies based on the Cancer and Aging Research Group Chemotherapy Toxicity Tool (CARG). Safety assessments were performed at each visit, and the severity of adverse events (AEs) was graded as per National Cancer Institute Common Terminology Criteria for Adverse Events (CTCAE) version 5.0. Dose modifications were allowed after the occurrence and resolution of grade 4 haematological or grade 2–4 non-haematological toxic effects. When patients experienced more than one type of grade 1 toxicity, the dose was proactively reduced to prevent worsening and ensure completion of the planned treatment.

Patients were classified into “toxicity” and “no toxicity” groups based on whether they experienced adverse effects related to treatment that required dose reduction (toxicity group) or not (no toxicity group) during at least the first six cycles of chemotherapy. Dose reductions are always protocolized. Dose reductions were implemented in cases of grade 2 or 3 non-haematological toxicity, or grade 1 toxicity, accompanied by multiple adverse events, following careful discussion and agreement with the patient. CTCAE grade ≥ 3 toxicity and especially non-haematological toxicity is extremely harmful for patients (e.g., diarrhoea (>7 stools a day) or asthenia (patients with previous ECOG PS 1 that present ECOG PS 4). To avoid it, and as recommended also in clinical trials [46,47,48], treatment can be held and/or dose-reduced when patients suffer grade 1–2 toxicities during therapy or at the time of patient evaluation, always at the physician’s discretion. In fact, this strategy allows patients to maintain dose intensity, preserving quality of life. Dose reductions were also made in cases of grade 4 haematological toxicity or grade 2 or 3 haematological toxicity that required more than one week for recovery. For instance, if there is haematological grade 2 toxicity (pe. < 1500 neutrophils or <75.000 platelets), treatment is held and a new evaluation is done 1 week later. Grade 1 or 2 diarrhoea, when associated with performance status deterioration, is also an indication to hold therapy until recovery (usually to grade 0 or 1). Dose reductions of 20% were done for haematological and non-haematological toxicities following clinical practice and national guidelines. Treatment withdrawal was adopted if patients required more than 2 dose reductions or due to patient–physician shared decisions.

### 4.3. DNA Isolation and Genotyping

To identify novel *DPYD* genetic variants and to assess the potential role of *TYMS*, *ENOSF1*, and *CDH4* variants in fluoropyrimidine toxicity, a subgroup of 256 samples was analysed using complementary approaches. These included targeted sequencing of the *DPYD* gene’s coding exons and *TYMS*, *ENOSF1*, and *CDH4* genetic variants analysed by RT-PCR. Full *DPYD* exon sequencing was analysed in 56 patients who were representative of the population. All analyses were conducted at the Laboratory of Pharmacogenomics, Department of Pharmacy, Hospital Gregorio Marañón, Madrid.

DNA was isolated from the peripheral whole blood of the patients included in the study using MagNA Pure Magnetic Glass Particle (MGP) Technology (Roche LifeScience, Rotkreuz, Switzerland) following the manufacturer’s instructions and was quantified using a Quawell 5000 spectrophotometer (Quawell Technology Inc., San Jose, CA, USA) after dilution to 10 ng/mL.

The *DPYD* gene was genotyped using real-time polymerase chain reaction (RT-PCR) with allele-specific TaqMan^®^ probes following the manufacturer’s instructions (Thermo Fisher Scientific, Waltham, MA, USA). The assay targets were clinically relevant single nucleotide polymorphisms (SNPs) associated with altered dihydropyrimidine dehydrogenase (DPD) activity, including DPYD*2A (rs3918290, c.1905+1G>A, IVS14+1G>A), DPYD*13 (rs55886062, c.1679T>G, I560S), *DPYD* c.2846A>T (rs67376798, D949V), and *DPYD* c.1129-5923C>G (rs75017182; haplotype B3). Each variant was detected using two differentially labelled Minor Groove Binder (MGB) TaqMan probes, providing high specificity and sensitivity for SNP discrimination. Genotyping was conducted using peripheral blood DNA samples, and results were interpreted according to current pharmacogenetic guidelines, including those from CPIC and DPWG [10].

Along with the identification of new variants, the following genetic polymorphisms were genotyped: *TYMS* (rs45445694, a polymorphic tandem repeat in the 5′UTR of the *TYMS* enhancer region (TSER), and rs11280056, a 9bp deletion 447 downstream of the *TYMS* transcription codon), *CDA* (rs2072671, c.79A>C, p.Lys27Gln), *ENOSF1* (rs2612091, NC_000018.10:g.683607C>T), and *CDH4* (rs6129058). *TYMS* variants were genotyped after PCR and amplicon length size analysis by electrophoresis as previously described [15]. *ENOSF1, CDH4* and *CDA* genetic variants were genotyped using TaqMan probes (C__15908768_10, C__30576234_10, C__25472931_20, respectively) (Applied Biosystems, Carlsbad, CA, USA) following the manufacturer’s recommendations.

The exonic regions of the *DPYD* gene were fully sequenced in a total of 56 patients (42 from the toxicity group and 14 from the control group) as previously described [23]. The resulting sequences were aligned and analysed using SnapGene software v8.2.2 (Dotmatics, Boston, MA, USA). 

SpliceAI [49] was used for predicting splice alterations caused by the variants found (https://spliceailookup.broadinstitute.org/, accessed on 1 September 2025). These tools also provide information from other predictive models such as PolyPhen (max), AlphaMissense [50], Combined Annotation Dependent Depletion (CADD) (https://cadd.gs.washington.edu/score, accessed on 1 September 2025), SIFT [51], PhyloP [52], PrimateAI-3 [53], REVEL, and Pangolin [54].

### 4.4. Three-Dimensional Modelling

The sequences of c.1863G>A, c.150+1G>A and wild-type DPD were exported from SnapGene v5.2 in Fasta format, and a 3D modelling of all of them was created using the RaptorX structure prediction server [55], accessed 22 August 2024. Protein database files were downloaded and visualised using Chimera [56] (University of California San Francisco, UCSF).

### 4.5. Statistical Analyses

Continuous clinical and demographic variables were expressed as the mean and standard deviation (SD) or as the median and interquartile range (IQR); qualitative variables were presented as absolute and relative frequencies. The chi-squared test (Fisher’s exact test, where appropriate) and *t*-test were used to compare qualitative and quantitative variables, respectively. Multivariate analyses were performed in significant univariate associations considering sex, type of fluoropyrimidine, Charlson Comorbidity Index, regimen and indication. The association between the selected genotypes and the time-to-reduction or withdrawal of fluoropyrimidine was analysed using Kaplan–Meier curves. For SNPs with a *p* value < 0.07, the hazard ratios (HR) adjusted using Cox regression were calculated based on sex, fluoropyrimidine and Charlson Comorbidity Index or sex, fluoropyrimidine type, Charlson Comorbidity Index, regimen and indication, with a 95% confidence interval (CI). A *p*-value < 0.05 was considered statistically significant. All data were analysed using IBM SPSS Statistics v.21 (IBM Corp., Armonk, New York, NY, USA).

## 5. Conclusions

In this real-world study, a substantial proportion of *DPYD* wild-type patients receiving standard fluoropyrimidine dosing experienced grade 2 (31%) or grade 3–4 (7%) toxicity, underscoring the limitations of current genotyping strategies in fully predicting adverse reactions.

Our findings highlight the clinical relevance of rare *DPYD* variants such as *c.150+1G>A* and *c.1863G>A* (p.Trp621Ter). These variants can result in severely impaired or truncated DPD enzyme function, significantly increasing the risk of life-threatening toxicity. Incorporating these variants into routine pre-emptive testing, after prospective validation, should help to identify high-risk individuals and guide appropriate dose adjustments, thereby improving treatment safety.

Moreover, we propose that additional genetic markers—such as DPYD*6 (c.2194G>A), and *DPYD c.496A>G*—should be systematically evaluated in prospective clinical trials. Variants in other genes involved in fluoropyrimidine metabolism, including *ENOSF1* rs2612091 and polymorphisms in *TYMS*, also warrant further investigation.

Integrating these emerging biomarkers into clinical trial designs will enable the generation of robust evidence regarding their predictive value. Ultimately, this could support their inclusion in future pharmacogenetic guidelines, enhancing the personalisation, safety, and efficacy of fluoropyrimidine-based chemotherapy.

## Figures and Tables

**Table 1:** Patient characteristics.

	Total (n = 256)	Toxicity (n = 117)	Not Toxicity (n = 139)	*p* Value
Sex				0.802
Men	124 (48.4%)	58 (49.6%)	66 (47.5%)	
Women	132 (51.6%)	59 (50.4%)	73 (52.5%)	
Age, median, IQR (min-max)	66, 16 (17–88)	66, 15 (31–88)	66, 17 (17–88)	0.321
Fluoropyrimidine				0.358
Capecitabine, n (%)	89 (34.8%)	37 (31.6%)	52 (37.4%)	
5-Fluorouracile, n (%)	167 (65.2%)	80 (68.4%)	87 (62.6%)	
Regimen				0.099
Monotherapy, n (%)	104 (40.6%)	40 (34.2%)	64 (46%)	
Double, n (%)	126 (49.2%)	64 (54.7%)	62 (44.6%)	
Triple, n (%)	26 (10.2%)	13 (11.1%)	13 (9.4%)	
Cancer type				
Gastrointestinal, n (%)	217 (84.8%)	102 (87.2%)	115 (82.7%)	0.384
Breast, n (%)	39 (15.2%)	15 (12.8%)	24 (17.3%)	
Indication				
Neoadjuvant, n (%)	64 (25%)	24 (20.5%)	40 (28.8%)	0.089
First line, n (%)	71 (27.7%)	33 (28.2%)	38 (27.3%)	
Second or successive lines, n (%)	39 (15.2%)	16 (13.6%)	23 (16.5%)	
Adjuvant, n (%)	82 (32%)	44 (37.6%)	38 (27.3%)	
Charlson Comorbidity Index (CCI)				
2	209 (81.6%)	96 (82.1%)	113 (81.3%)	1
>2	47 (18.4%)	21 (17.9%)	26 (18.7%)	

**Table 2:** Association of different genotypes with toxicity or dose reduction.

Gene	SNP	Genotype	n Toxicity (%)	*p* Value	adj *p* Value
*ENOSF1* *	rs2612019	TT (ref) (n = 75)	28 (37.3%)	0.041	
		TC (n = 127)	59 (46.5%)		TT vs. TC 0.242, 1.424 (0.788–2.575)
		CC (n = 54)	30 (55.6%)		TT vs. CC 0.049, 2.063 (1.000–4.257)
*TYMS* **	rs11280056	ins/ins (ref) (n = 72)	41 (56.9%)	0.092	
		ins/del (n = 88)	35 (39.8%)		ins/ins vs. ins/del 0.035, 0.498 (0.261–0.951)
		del/del (n = 6)	3 (50.0%)		ins/ins vs. del/del 0.887, 0.884 (0.160–4.881)
		ins/ins (ref) (n = 72) vs.	41 (56.9%)	0.042	0.042, 0.515 (0.272–0.975)
		ins/del + del/del (n = 94)	38 (40.4%)		

* Comparison of the whole group (n = 256); ** Comparison of the 5-fluorouracil treated group (n = 167).

**Table 3:** Association of different genotypes with withdrawal of patients.

Gene	SNP	Genotype	n Withdrawal (%)	*p* Value	adj *p* Value
*ENOSF1* *	rs2612019	TT (ref) (n = 75)	2 (2.7%)	0.008	TT vs. TC 0.042, 4.799 (1.055–21.825) TT vs. CC 0.016, 7.040 (1.434–34.564)
		TC (n = 127)	15 (11.8%)		
		CC (n = 54)	9 (16.7%)		
		TT (ref) (n = 75)	2 (2.7%)	0.011	0.025; 5.441 (1.239–23.885)
		TC+CC (n = 181)	24 (13.3%)		
*ENOSF1* **	rs2612019	TT (ref) (n = 53)	0 (0%)	0.013	NA
		TC (n = 82)	9 (11%)		
		CC (n = 32)	4 (12.5%)		
		TT (ref) (n = 53)	0 (0%)	0.010	NA
		TC+CC (n = 114)	13 (11.4%)		

* Comparison of the whole group (n = 256); ** Comparison of the 5-fluorouracil treated group (n = 167). NA: It is not possible to calculate the OR because there are no cases in any of the categories.

**Table 4:** Genetic variants found in patients who underwent *DPYD* sequencing.

dbSNP	Nucleotide Change	Protein Change	Exon/Intron	MAF Control	MAF * Toxicity
rs1801265	c.85T>C	p.Cys29Arg	Exon 2	0.1071	0.1547
rs37535663	c.150+1G>A	Splicing	Intron 2–3	<0.0001	0.0119
rs76210229	c.218T>G	p.Leu73Arg	Exon 3	<0.0001	0.0119
rs2297595	c.496A>G	p.Met166Val	Exon 6	0.1428	0.0595
rs13625776	c.515G>A	p.Arg172Lys	Exon 6	<0.0001	0.0119
rs45589337	c.775A>G	p.Lys259Glu	Exon 8	<0.0001	0.0119
rs56293913	c.1129-15T>C	Splicing	Intron 10–11	0.1428	0.0595
rs61622928	c.1218G>A	p.Met406Ile	Exon 11	<0.0001	0.0119
rs1801158	c.1601G>A	p.Ser534Asn	Exon 13	0.0714	0.0476
rs1801159	c.1627A>G	p.Ile543Val	Exon 13	0.1785	0.2500
rs1057516388	c.1863G>A	p.Trp621Ter	Exon 14	<0.0001	0.0119
rs1801160	c.2194G>A	p.Val732Ile	Exon 18	<0.0001	0.0952
rs114096998	c.3067C>A	p.Pro1023Thr	Exon 23	0.0357	<0.0001

* MAF, minor allele frequency.

## Data Availability

The original contributions presented in this study are included in this article/Supplementary Materials. Further inquiries can be directed to the corresponding authors.

## Supplementary Materials

The following supporting information can be downloaded at: https://www.mdpi.com/article/10.3390/ph19030460/s1, Table S1: Comorbidity scoring using the abbreviated Charlson Comorbidity Index. Table S2: Adverse events observed in the toxicity group and by tumour subtype. Table S3: Association of genotypes with toxicity or dose reduction. Table S4: Association of genotypes with toxicity or dose reduction (Capecitabine). Table S5: Association of genotypes with toxicity or dose reduction (5FU). Table S6: Association of genotypes with treatment withdrawal. Table S7: Association of genotypes with treatment withdrawal (Capecitabine), Table S8: Association of genotypes with treatment withdrawal (5FU).

## References

[1] Amstutz U., Farese S., Aebi S., Largiadèr C.R. (2009). Dihydropyrimidine dehydrogenase gene variation and severe 5-fluorouracil toxicity: A haplotype assessment. Pharmacogenomics.

[2] Boige V., Vincent M., Alexandre P., Tejpar S., Landolfi S., Le Malicot K., Greil R., Cuyle P.J., Yilmaz M., Faroux R. (2016). *DPYD* Genotyping to Predict Adverse Events Following Treatment With Fluorouracil-Based Adjuvant Chemotherapy in Patients with Stage III Colon Cancer: A Secondary Analysis of the PETACC-8 Randomized Clinical Trial. JAMA Oncol..

[3] Raida M., Schwabe W., Häusler P., Van Kuilenburg A.B., Van Gennip A.H., Behnke D., Höffken K. (2001). Prevalence of a common point mutation in the dihydropyrimidine dehydrogenase (DPD) gene within the 5’-splice donor site of intron 14 in patients with severe 5-fluorouracil (5-FU)- related toxicity compared with controls. Clin. Cancer Res..

[4] Del Re M., Michelucci A., Di Leo A., Cantore M., Bordonaro R., Simi P., Danesi R. (2015). Discovery of novel mutations in the dihydropyrimidine dehydrogenase gene associated with toxicity of fluoropyrimidines and viewpoint on preemptive pharmacogenetic screening in patients. EPMA J..

[5] Gentile G., Botticelli A., Lionetto L., Mazzuca F., Simmaco M., Marchetti P., Borro M. (2016). Genotype-phenotype correlations in 5-fluorouracil metabolism: A candidate *DPYD* haplotype to improve toxicity prediction. Pharmacogenom. J..

[6] Henricks L.M., Lunenburg C.A., Meulendijks D., Gelderblom H., Cats A., Swen J.J., Schellens J.H., Guchelaar H.-J. (2015). Translating *DPYD* genotype into DPD phenotype: Using the *DPYD* gene activity score. Pharmacogenomics.

[7] EMA Recommendations on DPD Testing Prior to Treatment with Fluorouracil, Capecitabine, Tegafur and Flucytosine. https://www.ema.europa.eu/en/news/ema-recommendations-dpd-testing-prior-treatment-fluorouracil-capecitabine-tegafur-and-flucytosine.

[8] Henricks L.M., Lunenburg C.A.T.C., de Man F.M., Meulendijks D., Frederix G.W.J., Kienhuis E., Creemers G.-J., Baars A., Dezentjé V.O., Imholz A.L.T. (2018). *DPYD* genotype-guided dose individualisation of fluoropyrimidine therapy in patients with cancer: A prospective safety analysis. Lancet Oncol..

[9] García-Alfonso P., Saiz-Rodríguez M., Mondéjar R., Salazar J., Páez D., Borobia A.M., Safont M.J., García-García I., Colomer R., García-González X. (2022). Consensus of experts from the Spanish Pharmacogenetics and Pharmacogenomics Society and the Spanish Society of Medical Oncology for the genotyping of *DPYD* in cancer patients who are candidates for treatment with fluoropyrimidines. Clin. Transl. Oncol..

[10] Pratt V.M., Cavallari L.H., Fulmer M.L., Gaedigk A., Hachad H., Ji Y., Kalman L.V., Ly R.C., Moyer A.M., Scott S.A. (2024). *DPYD* Genotyping Recommendations. J. Mol. Diagn..

[11] Lunenburg C.A.T.C., van der Wouden C.H., Nijenhuis M., Rhenen M.H.C.-V., de Boer-Veger N.J., Buunk A.M., Houwink E.J.F., Mulder H., Rongen G.A., van Schaik R.H.N. (2020). Dutch Pharmacogenetics Working Group (DPWG) guideline for the gene-drug interaction of *DPYD* and fluoropyrimidines. Eur. J. Hum. Genet..

[12] Amstutz U., Henricks L.M., Offer S.M., Barbarino J., Schellens J.H., Swen J.J., Klein T.E., McLeod H.L., Caudle K.E., Diasio R.B. (2018). Clinical Pharmacogenetics Implementation Consortium (CPIC) Guideline for Dihydropyrimidine Dehydrogenase Genotype and Fluoropyrimidine Dosing: 2017 Update. Clin. Pharmacol. Ther..

[13] ClinPGx Annotation of CPIC Guideline for Capecitabine and *DPYD* (Guideline Annotation PA166109594). https://www.clinpgx.org/guidelineAnnotation/PA166109594.

[14] Peeters S.L.J., Meulendijks D., Kadric Z., Ibrovic S., Creemers G., Milosevic V., van de Poll M., Simkens L.H.J., Deiman B.A.L.M., Gelderblom H. (2025). Real-world study on fluoropyrimidine-related toxicity outcomes in cancer patients with select *DPYD* variant alleles that received *DPYD* genotype-guided dosing. Int. J. Cancer.

[15] Rosmarin D., Palles C., Pagnamenta A., Kaur K., Pita G., Martin M., Domingo E., Jones A., Howarth K., Freeman-Mills L. (2015). A candidate gene study of capecitabine-related toxicity in colorectal cancer identifies new toxicity variants at *DPYD* and a putative role for *ENOSF1* rather than *TYMS*. Gut.

[16] Loganayagam A., Arenas Hernandez M., Corrigan A., Fairbanks L., Lewis C.M., Harper P., Maisey N., Ross P., Sanderson J.D., Marinaki A.M. (2013). Pharmacogenetic variants in the *DPYD*, *TYMS*, *CDA* and *MTHFR* genes are clinically significant predictors of fluoropyrimidine toxicity. Br. J. Cancer.

[17] García-González X., Cortejoso L., García M.I., García-Alfonso P., Robles L., Grávalos C., González-Haba E., Marta P., Sanjurjo M., López-Fernández L.A. (2015). Variants in *CDA* and *ABCB1* are predictors of capecitabine-related adverse reactions in colorectal cancer. Oncotarget.

[18] Ruiz-Pinto S., Pita G., Martín M., Nunez-Torres R., Cuadrado A., Shahbazi M.N., Caronia D., Kojic A., Moreno L.T., de la Torre-Montero J.C. (2021). Regulatory *CDH4* Genetic Variants Associate With Risk to Develop Capecitabine-Induced Hand-Foot Syndrome. Clin. Pharmacol. Ther..

[19] Johnson M.R., Wang K., Tillmanns S., Albin N., Diasio R.B. (1997). Structural organization of the human dihydropyrimidine dehydrogenase gene. Cancer Res..

[20] Richards S., Aziz N., Bale S., Bick D., Das S., Gastier-Foster J., Grody W.W., Hegde M., Lyon E., Spector E. (2015). Standards and guidelines for the interpretation of sequence variants: A joint consensus recommendation of the American College of Medical Genetics and Genomics and the Association for Molecular Pathology. Genet. Med..

[21] Borràs E., Dotor E., Arcusa A., Gamundi M.J., Hernan I., Dias M.D.S., Mañé B., Agúndez J.A.G., Blanca M., Carballo M. (2013). High-resolution melting analysis of the common c.1905+1G>A mutation causing dihydropyrimidine dehydrogenase deficiency and lethal 5-fluorouracil toxicity. Front. Genet..

[22] van Kuilenburg A.B., Meijer J., Maurer D., Dobritzsch D., Meinsma R., Los M., Knegt L.C., Zoetekouw L., Jansen R.L., Dezentjé V. (2017). Severe fluoropyrimidine toxicity due to novel and rare *DPYD* missense mutations, deletion and genomic amplification affecting DPD activity and mRNA splicing. Biochim. Biophys. Acta Mol. Basis Dis..

[23] García-González X., López-Tarruella S., García M.I., González-Haba E., Blanco C., Salvador-Martin S., Jerez Y., Thomas F., Jarama M., Sáez M.S. (2018). Severe toxicity to capecitabine due to a new variant at a donor splicing site in the dihydropyrimidine dehydrogenase *(DPYD)* gene. Cancer Manag. Res..

[24] Van Kuilenburg A.B., Meinsma R., Beke E., Bobba B., Boffi P., Enns G.M., Witt D.R., Dobritzsch D. (2005). Identification of three novel mutations in the dihydropyrimidine dehydrogenase gene associated with altered pre-mRNA splicing or protein function. Biol. Chem..

[25] Knikman J.E., Zhai Q., Lunenburg C.A.T.C., Henricks L.M., Böhringer S., van der Lee M., de Man F.M., Offer S.M., Shrestha S., Creemers G.-J. (2024). Discovering novel germline genetic variants linked to severe fluoropyrimidine-related toxicity in- and outside *DPYD*. Genome Med..

[26] Saarenheimo J., Wahid N., Eigeliene N., Ravi R., Salomons G.S., Ojeda M.F., Vijzelaar R., Jekunen A., van Kuilenburg A.B.P. (2021). Preemptive screening of *DPYD* as part of clinical practice: High prevalence of a novel exon 4 deletion in the Finnish population. Cancer Chemother. Pharmacol..

[27] Carter M.T., Nikkel S.M., Fernandez B.A., Marshall C., Noor A., Lionel A., Prasad A., Pinto D., Joseph-George A., Noakes C. (2011). Hemizygous deletions on chromosome 1p21.3 involving the *DPYD* gene in individuals with autism spectrum disorder. Clin. Genet..

[28] Malekkou A., Tomazou M., Mavrikiou G., Dionysiou M., Georgiou T., Papaevripidou I., Alexandrou A., Sismani C., Drousiotou A., Grafakou O. (2024). A novel large intragenic *DPYD* deletion causing dihydropyrimidine dehydrogenase deficiency: A case report. BMC Med. Genom..

[29] van Kuilenburg A.B., Meijer J., Mul A.N.P.M., Hennekam R.C.M., Hoovers J.M.N., de Die-Smulders C.E.M., Weber P., Mori A.C., Bierau J., Fowler B. (2009). Analysis of severely affected patients with dihydropyrimidine dehydrogenase deficiency reveals large intragenic rearrangements of *DPYD* and a de novo interstitial deletion del(1)(p13.3p21.3). Hum. Genet..

[30] Gaedigk A., Turner A.J., Moyer A.M., Zubiaur P., Boone E.C., Wang W.Y., Broeckel U., Kalman L.V. (2024). Characterization of Reference Materials for *DPYD*: A GeT-RM Collaborative Project. J. Mol. Diagn..

[31] Dobritzsch D., Schneider G., Schnackerz K.D., Lindqvist Y. (2001). Crystal structure of dihydropyrimidine dehydrogenase, a major determinant of the pharmacokinetics of the anti-cancer drug 5-fluorouracil. EMBO J..

[32] Smith M.M., Moran G.R. (2023). The unusual chemical sequences of mammalian dihydropyrimidine dehydrogenase revealed by transient-state analysis. Methods Enzymol..

[33] Xu B., Ionita-Laza I., Roos J.L., Boone B., Woodrick S., Sun Y., Levy S., A Gogos J., Karayiorgou M. (2012). De novo gene mutations highlight patterns of genetic and neural complexity in schizophrenia. Nat. Genet..

[34] Offer S.M., Fossum C.C., Wegner N.J., Stuflesser A.J., Butterfield G.L., Diasio R.B. (2014). Comparative functional analysis of *DPYD* variants of potential clinical relevance to dihydropyrimidine dehydrogenase activity. Cancer Res..

[35] Kim W., Cho Y.A., Kim D.C., Lee K.E. (2022). Elevated Risk of Fluoropyrimidine-Associated Toxicity in European Patients with *DPYD* Genetic Polymorphism: A Systematic Review and Meta-Analysis. J. Pers. Med..

[36] Del Re M., Cinieri S., Michelucci A., Salvadori S., Loupakis F., Schirripa M., Cremolini C., Crucitta S., Barbara C., Di Leo A. (2019). *DPYD**6 plays an important role in fluoropyrimidine toxicity in addition to *DPYD**2A and c.2846A>T: A comprehensive analysis in 1254 patients. Pharmacogenom. J..

[37] Božina N., Bilić I., Ganoci L., Šimičević L., Pleština S., Lešnjaković L., Trkulja V. (2022). *DPYD* polymorphisms c.496A>G, c.2194G>A and c.85T>C and risk of severe adverse drug reactions in patients treated with fluoropyrimidine-based protocols. Br. J. Clin. Pharmacol..

[38] García-González X., Kaczmarczyk B., Abarca-Zabalía J., Thomas F., García-Alfonso P., Robles L., Pachón V., Vaz Á., Salvador-Martín S., Sanjurjo-Sáez M. (2020). New *DPYD* variants causing DPD deficiency in patients treated with fluoropyrimidine. Cancer Chemother. Pharmacol..

[39] Ardizzone A., Bulzomì M., De Luca F., Silvestris N., Esposito E., Capra A.P. (2024). Dihydropyrimidine Dehydrogenase Polymorphism c.2194G>A Screening Is a Useful Tool for Decreasing Gastrointestinal and Hematological Adverse Drug Reaction Risk in Fluoropyrimidine-Treated Patients. Curr. Issues Mol. Biol..

[40] (2024). Raccomandazioni per Analisi Farmacogenetiche. https://www.aiom.it/2024-aiom-sif-raccomandazioni-per-analisi-farmacogenetiche/.

[41] Gross E., Ullrich T., Seck K., Mueller V., de Wit M., von Schilling C., Meindl A., Schmitt M., Kiechle M. (2003). Detailed analysis of five mutations in dihydropyrimidine dehydrogenase detected in cancer patients with 5-fluorouracil-related side effects. Hum. Mutat..

[42] De Luca O., Salerno G., De Bernardini D., Torre M.S., Simmaco M., Lionetto L., Gentile G., Borro M. (2022). Predicting Dihydropyrimidine Dehydrogenase Deficiency and Related 5-Fluorouracil Toxicity: Opportunities and Challenges of *DPYD* Exon Sequencing and the Role of Phenotyping Assays. Int. J. Mol. Sci..

[43] De Mattia E., Silvestri M., Polesel J., Ecca F., Mezzalira S., Scarabel L., Zhou Y., Roncato R., Lauschke V.M., Calza S. (2022). Rare genetic variant burden in *DPYD* predicts severe fluoropyrimidine-related toxicity risk. Biomed. Pharmacother..

[44] Hamzic S., Kummer D., Froehlich T.K., Joerger M., Aebi S., Palles C., Thomlinson I., Meulendijks D., Schellens J.H., García-González X. (2020). Evaluating the role of *ENOSF1* and *TYMS* variants as predictors in fluoropyrimidine-related toxicities: An IPD meta-analysis. Pharmacol. Res..

[45] Lecomte T., Ferraz J.M., Zinzindohoué F., Loriot M.-A., Tregouet D.-A., Landi B., Berger A., Cugnenc P.-H., Jian R., Beaune P. (2004). Thymidylate synthase gene polymorphism predicts toxicity in colorectal cancer patients receiving 5-fluorouracil-based chemotherapy. Clin. Cancer Res..

[46] Bridgewater J.A., Pugh S.A., Maishman T., Eminton Z., Mellor J., Whitehead A., Stanton L., Radford M., Corkhill A., O Griffiths G. (2020). Systemic chemotherapy with or without cetuximab in patients with resectable colorectal liver metastasis (New EPOC): Long-term results of a multicentre, randomised, controlled, phase 3 trial. Lancet Oncol..

[47] Amatu A., Patelli G., Zampino M.G., Bergamo F., Mosconi S., Tosi F., Ciardiello D., Lonardi S., Negrini G., Sibio D. (2025). Total neoadjuvant therapy followed by non-operative management or surgery in stage II-III rectal cancer (NO-CUT): A multicentre, single-arm, phase 2 trial. Lancet Oncol..

[48] Bond M.J.G., Bolhuis K., Loosveld O.J.L., de Groot J.W.B., Droogendijk H., Helgason H.H., Hendriks M.P., Klaase J.M., Kazemier G., Liem M.S.L. (2023). First-line systemic treatment strategies in patients with initially unresectable colorectal cancer liver metastases (CAIRO5): An open-label, multicentre, randomised, controlled, phase 3 study from the Dutch Colorectal Cancer Group. Lancet Oncol..

[49] Jaganathan K., Kyriazopoulou Panagiotopoulou S., McRae J.F., Darbandi S.F., Knowles D., Li Y.I., Kosmicki J.A., Arbelaez J., Cui W., Schwartz G.B. (2019). Predicting Splicing from Primary Sequence with Deep Learning. Cell.

[50] Cheng J., Novati G., Pan J., Bycroft C., Žemgulytė A., Applebaum T., Pritzel A., Wong L.H., Zielinski M., Sargeant T. (2023). Accurate proteome-wide missense variant effect prediction with AlphaMissense. Science.

[51] Ng P.C., Henikoff S. (2003). SIFT: Predicting amino acid changes that affect protein function. Nucleic Acids Res..

[52] Pollard K.S., Hubisz M.J., Rosenbloom K.R., Siepel A. (2010). Detection of nonneutral substitution rates on mammalian phylogenies. Genome Res..

[53] Gao H., Hamp T., Ede J., Schraiber J.G., McRae J., Singer-Berk M., Yang Y., Dietrich A.S.D., Fiziev P.P., Kuderna L.F.K. (2023). The landscape of tolerated genetic variation in humans and primates. Science.

[54] Zeng T., Li Y.I. (2022). Predicting RNA splicing from DNA sequence using Pangolin. Genome Biol..

[55] Källberg M., Margaryan G., Wang S., Ma J., Xu J. (2014). RaptorX server: A resource for template-based protein structure modeling. Methods in Molecular Biology.

[56] Pettersen E.F., Goddard T.D., Huang C.C., Couch G.S., Greenblatt D.M., Meng E.C., Ferrin T.E. (2004). UCSF Chimera—A visualization system for exploratory research and analysis. J. Comput. Chem..

