# Supplementary material for: Genetic Variants Associated with Fluoropyrimidine-Induced Toxicity in Real-World Patients After Pre-Emptive DPYD Pharmacogenetic Testing
Source: Pharmaceuticals (Basel). 2026 Mar 11;19(3):460. doi: 10.3390/ph19030460 (PMC13029575; doi:10.3390/ph19030460)
Supplement: Supplementary file 1 [file pharmaceuticals-19-00460-s001.zip › pharmaceuticals-4139932-supplementary.pdf]

Supplementary material

Comorbidity	Score
Cerebrovascular disease	1
Diabetes	1
Chronic obstructive pulmonary disease	1
Heart failure / ischemic heart disease	1
Dementia	1
Peripheral arterial disease	1
Chronic kidney failure (dialysis)	2
Cancer	2

Table S1. Comorbidity scoring using the abbreviated Charlson Comorbidity Index.

	**Total toxicity group (n=117)**	**Colorectal cancer (n=66)**	**Breast Cancer (n=17)**
**Asthenia**	43%	42%	12%
**Gastrointestinal toxicity**	32%	33%	29%
**Haematological toxicity**	20%	14%	0%
**Hand–foot syndrome**	21%	21%	53%
**Mucositis**	7%	3%	6%
**Peripheral neuropathy**	6%	6%	0%
**Transaminitis**	6%	11%	0%

Table S2. Adverse events observed in the toxicity group and by tumour subtype. Note: Percentages represent the proportion of patients within each subgroup who experienced each adverse event. The colorectal and breast cancer subgroups do not account for the full toxicity cohort because additional patients had other cancer types not shown in the table. Because individual patients could experience multiple adverse events, the percentages reported for each event do not sum to 100%.

Gene	SNP	Genotype	n (%)	*p* value	Adj *p* value, OR (95% IC)
CDA	rs2072671	AA (ref) (n=106)	50 (47.2%)		
		AC (n=119)	50 (42.0%)	0.852	AA vs AC 0.466, 0.820 (0.480-1.399)
		CC (n=31)	17 (54.8%)		AA vs CC 0.429, 1.338 (0.617-3.123)
		AA+AC (ref) vs CC		0.337	0.272, 1.535 (0.714-3.298)
		AA (ref) vs AC+CC		0.704	0.740, 0.918 (0.555-1.520)
ENOSF1	rs2612019	TT (ref) (n=75)	28 (37.3%)		
		TC (n=127)	59 (46.5%)	**0.041**	TT vs TC 0.206, 1.466 (0.811-2.652)
		CC (n=54)	30 (55.6%)		**TT vs CC 0.045, 2.094 (1.017-4.312)**
		TT+TC(ref) vs CC		0.124	0.113, 1.641 (0.890-3.028)
		TT(ref) vs TC+CC		0.098	0.087, 1.631 (0.932-2.855)
CDH4	rs6129058	GG (ref) (n=160)	71 (44.4%)		
		GT (n=80)	38 (47.5%)	0.608	GG vs GT 0.675, 1.123 (0.653-1.933)
		TT (n=16)	8 (50%)		GG vs TT 0.693, 1.232 (0.438-3.464)
		GG+GT (ref) vs TT		0.798	0.744, 1.185 (0.428-3.279)
		GG (ref) vs GT+TT		0.606	0.614, 1.141 (0.684-1.902)
TYMS	rs45445694 n=257	3R/3R (ref) (n=72)	30 (41.7%)		
		3R/2R (n=132)	61 (46.2%)	0.414	3R/3R vs 3R/2R 0.533, 1.207 (0.669-2.177)
		2R/2R (n=51)	25 (49%)		3R/3R vs 2R/2R 0.481, 1.304 (0.624-2.724)
		3R/3R+3R/2R (ref) vs 2R/2R			0.657, 1.153 (0.616-2.156)
		3R/3R(ref) vs 3R/2R+2R/2R		0.486	0.466, 1.223 (0.703-2.162)
	rs11280056 n=256	Ins/Ins (ref) (n=108)	56 (51.9%)		
		Ins/Del (n=133)	53 (39.8%)	0.235	ins/ins vs Ins/del 0.070, 0.620 (0.369-1.040)
		Del/Del (n=13)	7 (53.8%)		Ins/Ins vs Del/Del 0.812, 1.152 (0.359-3.696)
		Ins/Ins+Ins/Del (ref) vs Del/Del		0.579	0.482, 1.500 (0.484-4.645)
		Ins/Ins (ref) vs Ins/Del+Del/Del		0.099	0.102, 0.656 (0.395-1.087)

Table S3. Association of genotypes with toxicity or dose reduction.

Gene	SNP	Genotype	n (%)	*p* value	Adj *p* value, OR (95% IC)
CDA	rs2072671	AA (ref) (n=38)	17 (44.7%)		
	data	AC (n=41)	13 (31.7%)	0.083	AA vs AC 0.222, 0.551 (0.212-1.435)
		CC (n=10)	7 (70%)		AA vs CC 0.192, 2.807 (0.596-13.211)
	data	AA+AC (ref) vs CC		0.086	0.068, 3.893 (0.903-16.780)
		AA (ref) vs AC+CC		0.666	0.538, 0.755 (0.309-1.845)
ENOSF1	rs2612019	TT (ref) (n=22)	7 (31.8%)		
		TC (n=45)	19 (42.2%)	0.287	TT vs TC 0.529, 1.420 (0.477-4.225)
		CC (n=22)	11 (50.0%)		TT vs CC 0.358, 1.836 (0.503-6.700)
		TT+TC(ref) vs CC		0.456	0.492, 1.448 (0.504-4.160)
		TT(ref) vs TC+CC		0.327	0.419, 1.534 (0.544-4.326)
CDH4	rs6129058	GG (ref) (n=56)	21 (37.5%)		
		GT (n=29)	15 (51,7%)	0.417	GG vs GT 0.175, 1.921 (0.747-4.936)
		TT GT (n=4)	1 (25%)		GG vs TT 0.611, 0.544 (0.052-5.688)
		GG+GT (ref) vs TT		0.638	0.474, 0.428 (0.042-4.361)
		GG (ref) vs GT+TT		0.375	0.272, 1.660 (0.672-4.101)
TYMS n=257	rs45445694	3R/3R (ref) (n=22)	8 (36.4%)		
		3R/2R (n=49)	20 (40.8%)	0.427	3R/3R vs 3R/2R 0.813, 1.137 (0.393-3.294)
		2R/2R (n=18)	9 (50%)		3R/3R vs 2R/2R 0.617, 1.434 (0.349-5.896)
		3R/3R+3R/2R (ref) vs 2R/2R		0.435	0.657, 1.302 (0.405-4.187)
		3R/3R(ref) vs 3R/2R+2R/2R		0.625	0.742, 1.190 (0.422-3.355)
	rs11280056	WT (ref) (n=36)	15 (41.7%)		
		WT DEL (n=45)	18 (40.0%)	0.761	ins/ins vs Ins/del 0.934, 0.962 (0.380-2.432)
		DEL DEL (n=7)	4 (57,1%)		Ins/Ins vs Del/Del 0.427, 1.966 (0.371-10.403)
		Ins/Ins+Ins/Del (ref) vs Del/Del		0.448	0.386, 2.010 (0.414-9.759)
		Ins/Ins (ref) vs Ins/Del+Del/Del		1.0	0.617, 1.319 (0.445-3.909)

Table S4. Association of genotypes with toxicity or dose reduction (Capecitabine).

Gene	SNP	Genotype	n (%)	*p* value	Adj *p* value, OR (95% IC)
CDA	rs2072671	AA (ref) (n=68)	33 (48.5%)		
		AC (n=78)	37 (47.4%)	1	AA vs AC 0.935, 0.973 (0.504-1.879)
		CC (n=21)	10 (47.6%)		AA vs CC 0.959, 0.975 (0.364-2.612)
		AA+AC (ref) vs CC		1	0.980, 0.988 (0.391-2.498)
		AA (ref) vs AC+CC		1	0.716, 0.9887 (0.466-1.691)
ENOSF1	rs2612019	TT (ref) (n=53)	21 (39.6%)		
		TC (n=82)	40 (48.8%)	0.079	TT vs TC 0.287, 1.469 (0.724-2.984)
		CC (n=32)	19 (59.4%)		**TT vs CC 0.075, 2.626 (0.922-5.547)**
		TT+TC(ref) vs CC		0.171	0.145, 1.795 (0.817-3.942)
		TT(ref) vs TC+CC		0.183	0.135, 1.663 (0.854-3.240)
CDH4	rs6129058	GG (ref) (n=104)	50 (48.1%)		
		GT (n=51)	23 (45.1%)	0.806	GG vs GT 0.682, 0.868 (0.442-1.705)
		TT (n=12)	7 (58.3%)		GG vs TT 0.501, 1.517 (0.450-5.111)
		GG+GT (ref) vs TT		0.554	0.447, 1.589 (0.481-5.246)
		GG (ref) vs GT+TT		1	0.913, 0.966 (0.515-1.810)
TYMS *n=257*	rs45445694	3R/3R (ref) (n=50)	22 (44.0%)	0.659	
		3R/2R (n=83)	41 (49.4%)	0.427	3R/3R vs 3R/2R 0.547, 1.245 (0.610-2.544)
		2R/2R (n=33)	16 (48.5%)		3R/3R vs 2R/2R 0.672, 1.211 (0.499-2.937)
		3R/3R+3R/2R (ref) vs 2R/2R		1	0.885, 1.058 (0.490-2.284)
		3R/3R(ref) vs 3R/2R+2R/2R		0.613	0.539, 1.235 (0.630-2.420)
	rs11280056	WT (ref) (n=72)	41 (56.9%)		
		WT DEL (n=88)	35 (39.8%)	**0.092**	**Ins/Ins vs Ins/Del 0.036, 0.508 (0.269-0.958)**
		DEL DEL (n=6)	3 (50.0%)		Ins/Ins vs Del/Del 0.754, 0.766 (0.144-4.059)
		Ins/Ins+Ins/Del (ref) vs Del/Del		1	0.906, 1.103 (0.216-5.645)
		Ins/Ins (ref) vs Ins/Del+Del/Del		**0.042**	**0.041, 0.522 (0.279-0.975)**

* Tables may have a footer.

Table S5. Association of genotypes with toxicity or dose reduction (5FU).

Gene	SNP	Genotype	n (%)	*p* value	Adj *p* value, OR (95% IC)
CDA	rs2072671	AA (ref) (n=106)	9 (8.5%)		
		AC (n=119)	14 (11.8%)	0.646	AA vs AC 0.447, 1.421 (0.574-3.516)
		CC (n=31)	3 (9.7%)		AA vs CC 0.609 1.446 (0.352-5.942)
		AA+AC (ref) vs CC		1	0.796, 1.118 (0.322-4.390)
		AA (ref) vs AC+CC		0.532	0.426, 1.425 (0.596-3.409)
ENOSF1	rs2612019	TT (ref) (n=75)	2 (2.7%)		
		TC (n=127)	15 (11.8)	**0.008**	**TT vs TC 0.035, 5.135 (1.119-23.563)**
		CC (n=54)	9 (16.7%)		**TT vs CC 0.024, 6.363 (1.280-31.637)**
		TT+TC(ref) vs CC		0.081	0.189, 1.836 (0.742-4.547)
		TT(ref) vs TC+CC		**0.011**	**0.024, 5.526 (1.249-24.453)**
CDH4	rs6129058	GG (ref) (n=160)	17 (10.6%)		
		GT (n=80)	7 (8.8%)	1	GG vs GT 0.748, 0.857 (0.334-2.199)
		TT (n=16)	2 (12.5%)		GG vs TT 0.675, 1.407 (0.285-6.939)
		GG+GT (ref) vs TT		0.798	0.626, 1.477 (0.307-7.105)
		GG (ref) vs GT+TT		0.833	0.887, 0.939 (0.394-2.239)
TYMS	rs45445694 n=257	3R/3R (ref) (n=72)	5 (6.9%)		
		3R/2R (n=132)	13 (9.8%)	0.226	3R/3R vs 3R/2R 0.723, 1.219 (0.408-3.643)
		2R/2R (n=51)	7 (13.7%)		3R/3R vs 2R/2R 0.467, 1.606 (0.448-5.756)
		3R/3R+3R/2R (ref) vs 2R/2R		0.430	0.513, 1.394 (0.515-3.769)
		3R/3R(ref) vs 3R/2R+2R/2R		0.365	0.608, 1.316 (0.461-3.761)
	rs11280056 n=256	Ins/Ins (ref) (n=108)	12 (11.1%)		
		Ins/Del (n=133)	12 (9%)	0.591	Ins/Ins vs Ins/Del 0.652, 0.819 (0.344-1.951)
		Del/Del (n=13)	1 (7.7%)		Ins/Ins vs Del/Del 0.701, 0.655 (0.076-5.684)
		Ins/Ins+Ins/Del (ref) vs Del/Del		1	0.771, 0.731 (0.088-6.034)
		Ins/Ins (ref) vs Ins/Del+Del/Del		0.671	0.616, 0.804 (0.343-1.884)

* Tables may have a footer.

Table S6. Association of genotypes with treatment withdrawal.

Gene	SNP	Genotype	n (%)	*p* value	Adj *p* value, OR (95% IC)
CDA	rs2072671	AA (ref) (n=38)	4 (10.5%)		
		AC (n=41)	7 (17.1%)	0.626	0.510, 1.606 (0.392-6.583)
		CC (n=10)	2 (20%)		0.366, 2.501 (0.343-18.220)
		AA+AC (ref) vs CC		0.636	0.485, 1.868 (0.324-10.788)
		AA (ref) vs AC+CC		0.384	0.420, 1.744 (0.451-6.740)
ENOSF 1	rs2612019	TT (ref) (n=22)	2 (9.1%)		
		TC (n=45)	6 (13.3%)	0.528	0.610, 1.576 (0.274-9.051)
		CC (n=22)	5 (22.7%)		0.451, 2.079 (0.310-13.918)
		TT+TC(ref) vs CC		0.295	0.564, 1.520 (0.367-6.296)
		TT(ref) vs TC+CC		0.506	0.511, 1.742 (0.332-9.133)
CDH4	rs6129058	GG (ref) (n=56)	8 (14.3%)		
		GT (n=29)	4 (13.8%)	0.752	0.657, 1.376 (0.337-5.625)
		TT GT (n=4)	1 (25%)		0.430, 2.698 (0.230-31.661)
		GG+GT (ref) vs TT		0.638	0.477, 2.380 (0.218-25.964)
		GG (ref) vs GT+TT		1	0.533, 1.527 (0.404-5.771)
TYMS n=257	rs45445694	3R/3R (ref) (n=22)	3 (13.6%)		
		3R/2R (n=49)	6 (12.2%)	0.608	0.543, 0.616 (0.129-2.9339)
		2R/2R (n=18)	4 (22.2%)		0.817, 0.796 (0.115-5.527)
		3R/3R+3R/2R (ref) vs 2R/2R		0.289	0.866, 1.145 (0.239-5.474)
		3R/3R(ref) vs 3R/2R+2R/2R		1	0.580, 0.654 (0.146-2.943
	rs11280056	WT (ref) (n=36)	6 (16.7%)		
		WT DEL (n=45)	6 (13.3%)	0.900	0.653, 0.736 (0.193-2.807)
		DEL DEL (n=7)	1 (14.3%)		0.904, 1.156 (0.109-12.313)
		Ins/Ins+Ins/Del (ref) vs Del/Del		1	0.786, 1.368 (0.143-13.109)
		Ins/Ins (ref) vs Ins/Del+Del/Del		0.764	0.709, 0.783 (0.216-2.834)

Table S7. Association of genotypes with treatment withdrawal (Capecitabine).

Gene	SNP	Genotype	n (%)	*p* value	Adj *p* value, OR (95% IC)
CDA	rs2072671	AA (ref) (n=68)	5 (7.4%)		
		AC (n=78)	7 (9.0%)	1	0.770, 1.198 (0.356-4.024)
		CC (n=21)	1 (4.8%)		0.730, 0.675 (0.073-6.278)
		AA+AC (ref) vs CC		1	0.653, 0.614 (0.073-5.155)
		AA (ref) vs AC+CC		1	0.888, 1.088 (0.337-3.512)
ENOSF 1	rs2612019	TT (ref) (n=53)	0 (0%)		
		TC (n=82)	9 (11.0%)	**0.013**	NA
		CC (n=32)	4 (12.5%)		NA
		TT+TC(ref) vs CC		0.277	0.225, 2.206 (0.615-7.911)
		TT(ref) vs TC+CC		**0.010**	NA
CDH4	rs6129058	GG (ref) (n=104)	9 (8.7%)		
		GT (n=51)	3 (5.9%)	0.901	0.533, 0.648 (0.166-2.534)
		TT (n=12)	1 (8.3%)		0.999, 1.001 (0.113-8.835)
		GG+GT (ref) vs TT		0.554	0.909, 1.134 (0.132-9.739)
		GG (ref) vs GT+TT		0.768	0.588, 0.711 (0.208-2.436)
TYMS n=257	rs45445694	3R/3R (ref) (n=50)	2 (4%)		
		3R/2R (n=83)	7 (8.4%)	0.622	0.412, 1.978 (0.388-10.093)
		2R/2R (n=33)	3 (9.1%)		0.351, 2.429 (0.376- 15.681)
		3R/3R+3R/2R (ref) vs 2R/2R		0.707	0.564, 1.510 (0.373-6.121)
		3R/3R(ref) vs 3R/2R+2R/2R		0.514	0.354, 2.099 (0.438-10.067)
	rs11280056	WT (ref) (n=72)	6 (8.3%)		
		WT DEL (n=88)	6 (6.8%)	0.854	0.742, 0.819 (0.250-2.687)
		DEL DEL (n=6)	0 (0%)		0.999, 0 (0-)
		Ins/Ins+Ins/Del (ref) vs Del/Del		1	0.999, 0.0 (0-)
		Ins/Ins (ref) vs Ins/Del+Del/Del		0.765	0.654. 0.763 (0.233-2.496)

Table S8. Association of genotypes with treatment withdrawal (5FU).
